# Distinct effects of a dairy probiotic drink and physical exercise on gut microbiota in pediatric acute lymphoblastic leukemia patients in remission: a randomized clinical trial

**DOI:** 10.3389/fmicb.2026.1791192

**Published:** 2026-04-13

**Authors:** Libuša Nechalová, Ivan Hric, Sabína Smahová, Miriam Babicová, Simona Ugrayová, Veronika Hlaváčová, Leonard Lendvorský, Timotej Surán, Alexandra Kolenová, Viktor Bielik

**Affiliations:** 1 Department of Biological and Medical Sciences, Faculty of Physical Education and Sports, Comenius University in Bratislava, Bratislava, Slovakia; 2 Biomedical Research Center, Institute of Clinical and Translational Research, Slovak Academy of Sciences, Bratislava, Slovakia; 3 Department of Pediatric Hematology and Oncology, Comenius University and National Institute of Children's Diseases, Bratislava, Slovakia; 4 National Sports Centre, Bratislava, Slovakia

**Keywords:** cancer, handgrip strength, *Lacticaseibacillus paracasei*, *Veillonella*, β diversity

## Abstract

**Introduction:**

Treatment of pediatric acute lymphoblastic leukemia (ALL) induces persistent gut microbiota dysbiosis. However, microbiota-targeted interventions remain limited. This study evaluated the independent effects of physical exercise and probiotic supplementation on gut microbiota composition in pediatric ALL patients in remission for <5 years.

**Methods:**

Thirty patients were randomized to an experimental group (8-week exercise followed by 8-week daily probiotic intake) or a non-intervention control group. Gut microbiota composition, stool metabolite profiles, anthropometric parameters, and physical strength outcomes were assessed.

**Results:**

Physical exercise improved handgrip strength and supported anthropometric stability, with only modest effects on gut microbiota composition. In contrast, probiotic supplementation induced a significant shift in gut microbial community structure permutational multivariate analysis of variance (PERMANOVA, *R*^2^ = 0.036, *p* = 0.041), accompanied by increases in lactic acid bacteria (LAB) and short-chain fatty acid (SCFA)–producing taxa.

**Discussion:**

Overall, probiotic supplementation had a greater impact on gut microbiota composition than physical activity, inducing targeted shifts toward a more favorable microbial profile in this clinically vulnerable population.

## 1 Introduction

Acute lymphoblastic leukemia (ALL) is the most common childhood cancer, with a pronounced early-life peak in incidence between the ages of 1 and 4 years (Marcotte et al., 2021). Treatment typically involves aggressive chemotherapy and prolonged antibiotic exposure, which can significantly disturb the diversity and functionality of the gut microbiota (Rajagopala et al., 2020). As a result, the microbial composition of ALL patients' exhibits reduced diversity and greater inter-individual dispersion compared with the compact clustering observed in healthy controls (Liu et al., 2020). Furthermore, gut dysbiosis in this population is characterized by a reduced abundance of short-chain fatty acid (SCFA)–producing taxa (Song and Gyarmati, 2024), an increased abundance of opportunistic pathogens, and alterations linked to antimicrobial resistance (Luo et al., 2024). These treatment-related shifts may persist long after therapy has ended and contribute to long-term health consequences, including metabolic disturbances (Bhuta et al., 2021), impaired physical fitness (Järvelä et al., 2010), and an increased risk of overweight and obesity (Ladas et al., 2025).

Probiotics are considered a cornerstone of gut microbiome rebiosis and modulation across different clinical study populations, including colorectal cancer (Gao et al., 2015), breast cancer (Pellegrini et al., 2020), and other oncological diseases (Wang et al., 2022). Although probiotics are widely regarded as effective microbiota modulators, their use in pediatric ALL has been investigated in only a limited number of studies, primarily as a strategy to mitigate chemotherapy-induced gastrointestinal side effects (Reyna-Figueroa et al., 2019). Consequently, microbiota-targeted approaches have not been systematically evaluated with their potential to improve gut microbiota composition or long-term health outcomes in pediatric ALL survivors, leaving a critical gap in post-treatment supportive care. Additionally, exercise-based interventions in ALL have primarily focused on the effects of physical activity on the skeletal, musculoskeletal, neuromuscular, cardiopulmonary, and cardiovascular systems, as well as on fatigue, balance disorders, and metabolic alterations (Simioni et al., 2018; Coombs et al., 2020; Liu et al., 2024). Nonetheless, a growing body of evidence links physical training and exercise to favorable changes in the structure and function of the gut microbiota (Pérez-Prieto et al., 2024), contributing to a healthier microbiome with benefits for overall human health (Donati Zeppa et al., 2021).

Accordingly, the aim of this study was to investigate the effects of an 8-week structured physical activity program followed by an 8-week probiotic supplementation on gut microbiota diversity and composition, physical fitness, and anthropometric parameters in pediatric ALL patients in early remission. Building on earlier findings, the present study aimed to distinguish the individual contributions of each intervention by evaluating them separately.

## 2 Materials and methods

### 2.1 Study subjects, recruitment, and design

**Figure 1:**
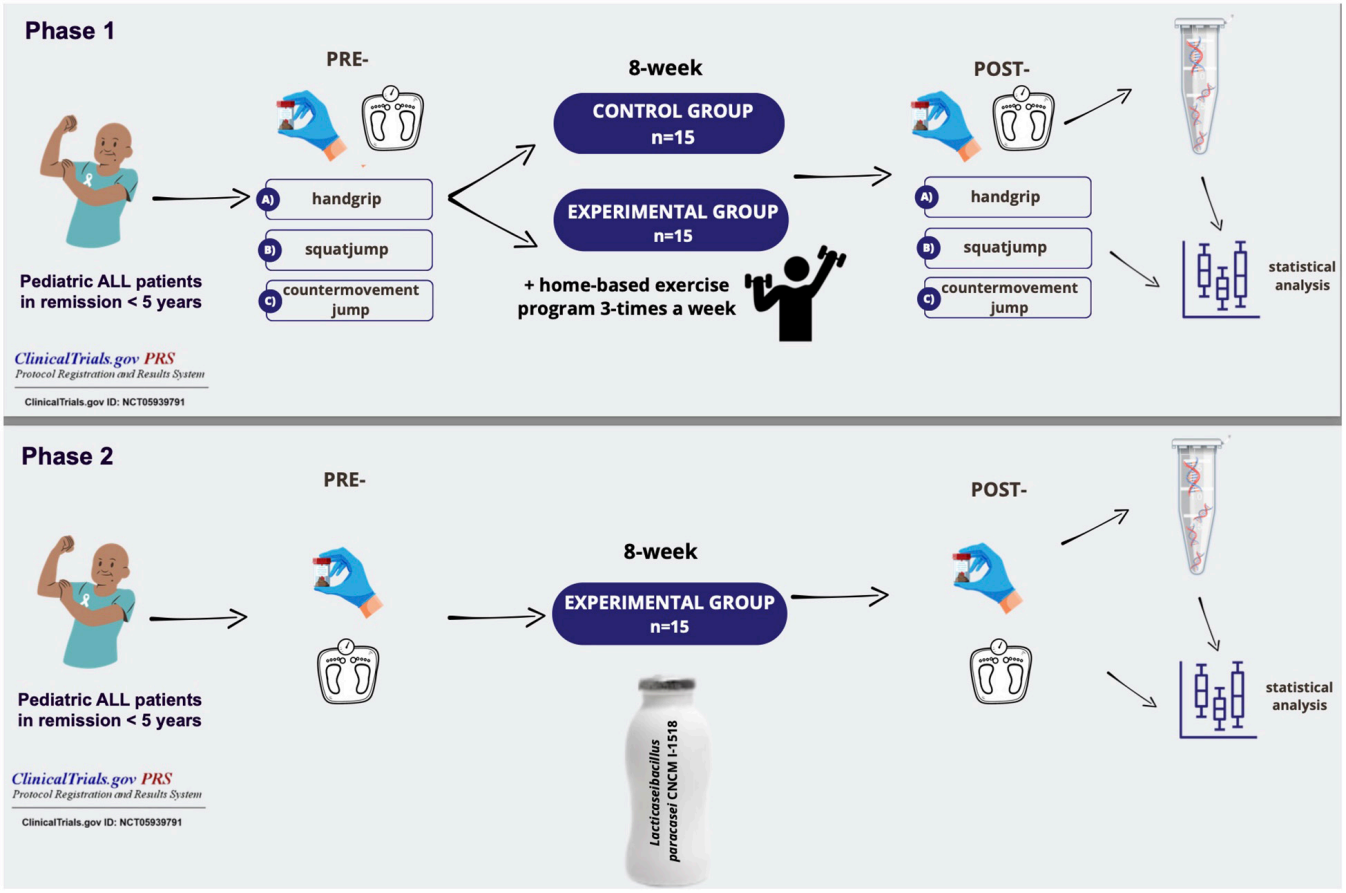
Overview of study design.

The study cohort included 30 pediatric patients previously diagnosed and treated for acute lymphoblastic leukemia (ALL), who had been in remission for < 5 years. All participants were aged between 6 and 15 years and were recruited from the Department of Pediatric Hematology at the National Institute of Children's Diseases (NICD) in Bratislava, Slovakia. Participants were randomly assigned to study groups using a computer-generated randomization sequence generated with an online randomization tool (https://www.randomizer.org). Participants were allocated into two groups: (a) an experimental group, which participated in both a physical exercise program (ExpPA) and a probiotic intervention (ExpACT), and (b) a non-intervention control group (Ctrl). The experimental group completed an 8-week physical exercise intervention (Phase 1), followed by a 4-week washout period before initiating an 8-week probiotic supplementation (Phase 2). Figure 1 illustrates the study design.

The study was performed between September 2023 and March 2024, in accordance with the principles outlined in the Declaration of Helsinki for research involving human subjects. The legal representatives of the participants provided informed consent after receiving a detailed explanation of the study procedures and discussing them with the investigators. Ethical approval was granted by the Ethics Committee of the National Institute of Children's Diseases (EK/1/22). Full clinical trial registration is available at ClinicalTrials.gov (NCT05939791).

### 2.2 Medical treatment

The patients were treated for B-cell acute lymphoblastic leukemia (B-ALL) according to the AIEOP-BFM ALL 2009 treatment protocol (EU Clinical Trials Register, 2025). Induction therapy included prednisone, anthracyclines, vincristine, and PEG-asparaginase. Early consolidation included cytarabine (araC), cyclophosphamide, and 6-mercaptopurine, followed by high-dose methotrexate during the consolidation phase. The intensification phase comprised dexamethasone, anthracyclines, vincristine, PEG-asparaginase, and intrathecal methotrexate. Maintenance therapy consisted of weekly methotrexate and daily 6-mercaptopurine.

### 2.3 Experimental intervention

#### 2.3.1 Phase 1: physical exercise program

The online physical exercise program delivered via MS Teams for the ExpPA consisted of 45–60 min of moderate- to vigorous-intensity physical exercise, supervised by a certified sports trainer from the Faculty of Physical Education and Sports at Comenius University. The program was adapted from our previous study (Bielik et al., 2023), with a training frequency of three sessions per week. The 8-week program was designed to enhance endurance and progressively rebuild muscular strength. Training sessions emphasized the activation of large muscle groups and the development of proper exercise technique. Each exercise included 10 to 15 repetitions per set, with a total of 2 to 3 sets. The exercises typically incorporated three modalities, arranged according to increasing difficulty. Exercise selection was tailored to each participant based on individual health status and fitness level. A physiotherapist from the Physiotherapy and Rehabilitation Department at NICD was consulted as needed prior to each session (Babicová et al., 2024).

#### 2.3.2 Phase 2: probiotic supplementation

Following the physical activity intervention, the experimental group underwent a 4-week washout period to minimize carryover effects and due to the Christmas holiday period. Thereafter, participants initiated daily consumption of a commercially available probiotic dairy product, previously used in our clinical study (Bielik et al., 2023) and selected due to its well-established safety profile, for a duration of 8 weeks. The study product was a commercially available, sweetened, flavored, fermented dairy drink (Actimel^®^) (Danone, Belgium) containing at least 2 × 10^10^ colony-forming units (CFU)/100 g of product (per bottle) of the strain *Lacticaseibacillus paracasei* subsp. *paracasei* CNCM I-1518, combined with a symbiosis of two cultures commonly used in yogurt, namely *Streptococcus thermophilus* and *Lactobacillus delbrueckii* subsp. *bulgaricus* (at least 10^9^ CFU/100 g for the whole symbiosis), and supplemented with vitamins D_3_ (2.07 μg/100 g) and B_6_ (0.3 mg/100 g).

### 2.4 Anthropometric and physical strength assessment

The examination was conducted at the National Sports Center in Bratislava, Slovakia. Anthropometric measurements included body height, body weight, and waist and hip circumferences. Body weight was measured using a calibrated TONAVA TH200 scale (Sklenár, s.r.o., Dlouhonovice, Czech Republic), while waist and hip circumferences were measured using a standardized, flexible tape. Body mass index (BMI) percentiles were derived from height and weight measurements, using Centers for Disease Control and Prevention reference calculators for children and adolescents aged 2–20 years (UpToDate^®^, sex-specific calculators; Calculator, 2025). Physical strength assessments included handgrip strength and vertical jump performance, conducted at baseline and after the physical exercise intervention. Handgrip strength was measured using a CAMRY dynamometer (model EH101, ISO 9001–certified). Participants stood upright with the dominant arm raised and were instructed to exert maximal grip strength while slowly lowering the arm to their side over a 5-s period. The highest value from three trials, each separated by a 15-s rest interval, was recorded. Vertical jump performance was evaluated using the squat jump (SJ) and countermovement jump (CMJ), assessed with the Optojump system (Microgate, Bolzano, Italy). Participants performed three trials of each jump type, with a 30-s rest between attempts. The highest jump height from each test was recorded for analysis.

### 2.5 Microbial analysis

The fecal samples were collected using standard stool collection tubes. Participants were instructed on proper collection procedures to minimize the risk of contamination. Total DNA was extracted from fecal samples using the ZymoBIOMICS DNA/RNA kit (Zymo Research, Irvine, CA, USA) according to the manufacturer's protocol. NGS libraries were prepared using the 16S Microbiome NGS Assay (ViennaLab Diagnostics GmbH, Vienna, Austria). In the first PCR step, the hypervariable V3–V4 regions of the 16S rRNA gene were amplified using locus-specific primers, and amplicons were verified by agarose gel electrophoresis. The second low-cycle PCR introduced dual indices for sample demultiplexing. Both PCR products were purified with Agencourt AMPure XP magnetic beads (Beckman Coulter, Brea, CA, USA). DNA libraries were quantified using a Qubit 2.0 Fluorometer (Thermo Fisher Scientific, Waltham, MA, USA), and fragment size distributions were verified using an Agilent 2100 Bioanalyzer (Agilent Technologies, Santa Clara, CA, USA) with a High Sensitivity DNA Kit (Agilent Technologies). DNA libraries were diluted to 2 nM, pooled equimolarly, and sequenced on the Illumina NextSeq 2000 platform (Illumina Inc., San Diego, CA, USA) using 300-bp paired-end reads.

### 2.6 Illumina data processing

Sequencing data were analyzed using the software provided with the ViennaLab NGS Microbiome Assay kit. The reads were preprocessed using BBMerge, Cutadapt, and SeqKit (Shen et al., 2016; Bushnell et al., 2017). Species-level read classification was performed using the CLARK sequence classification system (Ounit et al., 2015), which is based on discriminative k-mers in a sequence database. The reference databases used for classification were constructed from sequences in the SILVA (v138.1) and UNITE (v10) repositories (Quast et al., 2012; Nilsson et al., 2019). Taxonomic information was obtained from the NCBI taxonomy database (https://www.ncbi.nlm.nih.gov/taxonomy/).

### 2.7 Stool metabolites; NMR data acquisition

NMR data were acquired using an Avance III 600-MHz spectrometer equipped with a cryoprobe (Bruker, Ettlingen, Germany). Sample preparation, acquisition settings, and processing steps followed the analytical procedures described in our previous study (Hric et al., 2026). Briefly, stool samples were stored at 6 °C for no longer than 3 h prior to measurement, randomly ordered, and analyzed at 310 K using standard Bruker profiling protocols (1D NOESY, CPMG, COSY, and J-resolved). The concentrations of the following 21 stool metabolites were analyzed: propionate, acetate, butyrate, methanol, formate, lactate, threonine, leucine, isoleucine, valine, glycine, alanine, glutamate, aspartate, fumarate, phenylalanine, tyrosine, tryptophan, nicotinamide, succinate, and glutamine.

### 2.8 Statistical analysis

All statistical analyses were conducted in R (R Core Team, 2021) (version 4.5.0) using the vegan and phyloseq packages. Normality was assessed using the Shapiro–Wilk test. Prior to statistical analysis, microbiome data were filtered to minimize the influence of rare and low-abundance taxa: taxa occurring in fewer than 20% of samples or with a mean relative abundance below 0.001% were excluded. α diversity was calculated from the species-level abundance data using MOTHUR (v1.41.3) (Schloss et al., 2009). Alpha diversity of the gut microbiota was estimated with the Shannon and Chao1 indices. β diversity was calculated using the Bray–Curtis dissimilarity index and visualized via principal coordinate analysis (PCoA). Microbiota composition was evaluated using permutational multivariate analysis of variance (PERMANOVA) based on Bray–Curtis distances with 999 permutations, with permutations stratified by participant ID to account for the paired design. Differences in body composition, physical fitness tests, stool metabolite concentrations, gut diversity, and the relative abundance of specific bacterial taxa between time points and groups were analyzed using the two-tailed Wilcoxon signed-rank test and the Mann–Whitney U-test. Effect sizes for non-parametric comparisons were quantified using rank-biserial correlation (*r*_rβ_). Results from differential abundance analyses (DAA) were visualized using bubble plot. Statistical significance was set at *p* < 0.05. *P-values* are reported as unadjusted *p-values* and should be interpreted as exploratory. Based on our prior study in pediatric ALL survivors (Bielik et al., 2023) (mean Shannon diversity 3.22, SD ± 0.45), our study with 15 participants per group had 80% statistical power at α = 0.05 to detect a 15% increase in Shannon diversity following the intervention.

## 3 Results

### 3.1 Phase 1: physical exercise program

In the first phase, 10 of the 15 participants allocated to the ExpPA completed the physical exercise program. The remaining five participants were excluded due to insufficient attendance (< 80%). In the Ctrl, nine children completed the study, while six were excluded due to absence at follow-up assessments or incomplete sample collection.

#### 3.1.1 Anthropometric measurements and physical strength tests

Following the physical training intervention, a significant increase in body height and body weight was observed. Furthermore, a significant improvement was observed in handgrip strength. In the Ctrl, significant increases were detected in body height, waist circumference, and hip circumference, while no significant changes were found in physical strength measures. All significant within-group changes were associated with large effect sizes. A detailed overview of body composition and physical performance outcomes is presented in Table 1.

**Table 1:** Body composition and physical strength tests in both groups.

Variable	Differance ExpPA	Differance Ctrl	*P*-value			r_rβ_		
			Within ExpPA	Within Ctrl	Between groups	Within ExpPA	Within Ctrl	Between groups
Body height (cm)	1.25 ± 0.75	1.5 ± 1.34	**0.011**	**0.017**	0.934	1.000	1.000	0.022
Body weight (kg)	1.11 ± 1.10	0.28 ± 2.33	**0.008**	0.173	0.414	1.000	0.511	0.222
BMI percentil	2.85 ± 7.73	−3.62 ± 12.11	0.440	0.767	**0.008**	0.288	0.111	0.711
WC (cm)	−0.20 ± 2.94	2.44 ± 2.30	0.724	**0.027**	0.119	0.138	0.822	0.422
HC (cm)	1.6 ± 2.27	1.67 ± 3.40	0.072	**0.016**	0.306	0.636	1.000	0.277
Handgrip (N/kg)	30.43 ± 36.44	−4.36 ± 33.37	**0.017**	0.858	0.540	0.854	0.066	0.166
SJ (cm)	0.40 ± 2.43	−0.14 ± 2.09	0.645	0.888	0.414	0.163	0.055	0.222
CMJ (cm)	0.54 ± 1.87	−1.51 ± 2.54	0.444	0.075	0.567	0.272	0.666	0.155

Values are presented as mean ± standard error of the mean. Table summarizing significant differences (in bold) at *p* < 0.05 and effect size by rank-biserial correlation. r_rβ_, rank-biserial correlation; ExpPA, experimental group with physical exercise program; Ctrl, control group; BMI, body mass index; WC, waist circumference; HC, hip circumference; SJ, squat jump; CMJ, countermovement jump.

#### 3.1.2 Gut microbiota analysis

Following the physical training intervention, an increasing trend in α diversity was reported, whereas a decreasing trend was observed in the Ctrl. The Shannon and Chao1 indices increased in the ExpPA group, whereas both indices decreased in the Ctrl group. In parallel, β diversity analysis using PERMANOVA at the species level revealed no significant differences in the overall microbial community structure across groups and timepoints (*R*^2^ = 0.078, *p* = 0.946). Following the intervention, the ExpPA exhibited a modest but significant increases in the relative abundances of several beneficial SCFA-producing taxa, including *Anaerotignum lactatifermentans, Bacteroides cellulosilyticus, Coprococcus eutactus*, or *Prevotella rara*. No significant changes were observed between groups at baseline or within Ctrl for the aforementioned taxa. A detailed overview of bacterial composition and diversity indices is presented in Table 2.

**Table 2:** Gut microbiota composition, diversity indices, and metabolites in both groups during Phase 1: physical exercise program.

Variable	ExpPA		Ctrl		***P***-value		***r***_**rβ**_	
	Pre	Post	Pre	Post	Within ExpPA	Within Ctrl	Within ExpPA	Within Ctrl
Shannon index	3.120 ± 0.294	3.290 ± 0.233	3.311 ± 0.145	3.244 ± 0.410	0.057	0.750	0.711	0.143
Chao1 index	505.850 ± 78.774	552.200 ± 68.025	495.656 ± 83.706	495.022 ± 86.911	0.074	0.678	0.636	0.156
*Anaerotignum lactatifermentans*	0.009 ± 0.008	0.022 ± 0.014	0.051 ± 0.069	0.145 ± 0.315	**0.016**	0.859	0.854	0.067
*Bacteroides cellulosilyticus*	0.007 ± 0.022	0.158 ± 0.423	0.007 ± 0.021	0.090 ± 0.229	**0.028**	0.109	0.929	1.000
*Coprococcus eutactus*	0.001 ± 0.002	0.007 ± 0.013	0.026 ± 0.063	0.031 ± 0.070	**0.028**	0.779	0.929	0.111
*Prevotella rara*	0.000 ± 0.001	0.324 ± 1.016	0.436 ± 1.307	0.127 ± 0.378	**0.043**	0.715	1.000	0.200
Trimethylamine	3.89E-04 ± 2.32E-04	3.41E-04 ± 2.14E-04	4.40E-04 ± 3.74E-04	3.33E-04 ± 1.92E-04	**0.047**	0.678	0.709	0.156
Propionate	1.99E-02 ± 7.57E-03	1.94E-02 ± 5.83E-03	2.64E-02 ± 7.35E-03	2.00E-02 ± 4.89E-03	0.878	**0.028**	0.055	0.822
Methanol	1.25E-03 ± 4.51E-04	1.33E-03 ± 4.82E-04	9.78E-04 ± 4.11E-04	3.17E-03 ± 2.65E-03	0.721	**0.038**	0.127	0.778

Values are presented as mean ± standard error of the mean. Table summarizing significant differences (in bold) at *p* < 0.05 and effect size by rank-biserial correlation. r_rβ_, rank-biserial correlation; ExpPA, experimental group with physical exercise program; Ctrl, control group.

#### 3.1.3 Stool metabolites

In the ExpPA, a significant decrease in trimethylamine (TMA) was observed. In the Ctrl, a significant decrease in propionate was detected, along with significant increase in methanol. A detailed overview of bacterial metabolites is presented in Table 2. Correlational analyses revealed a significant negative association between *Actinomycetota* and trimethylamine (*r* = −0.489, *p* = 0.033), as well as between *Bifidobacterium* and trimethylamine (*r* = −0.478, *p* = 0.038). Moreover, a significant positive correlation was identified between butyrate and *Faecalibacterium* (*r* = 0.521, *p* = 0.022).

### 3.2 Phase 2: probiotic supplementation

In the second phase, 14 of the 15 children in the ExpACT completed the 8-week dairy probiotic intervention. The remaining one participant was excluded due to non-compliance with the probiotic supplementation protocol.

#### 3.2.1 Anthropometric measurements

After 8-week probiotic intervention a significant increase in both body height and body weight was observed. Body height increased from 137.6 ± 18.27 cm to 139.54 ± 18.55 cm (*p* = 0.001, *r*_rβ_ = 0.962), while body weight increased from 34.6 ± 12.13 kg to 36.16 ± 12.95 kg (*p* = 0.001, *r*_rβ_ = 0.1). However, no significant change was detected in BMI percentile (ExpACT-pre 56.5 ± 30.2, ExpACT-post 56.6 ± 30.2, *p* = 0.952).

#### 3.2.2 Gut microbiota analysis

**Figure 2:**
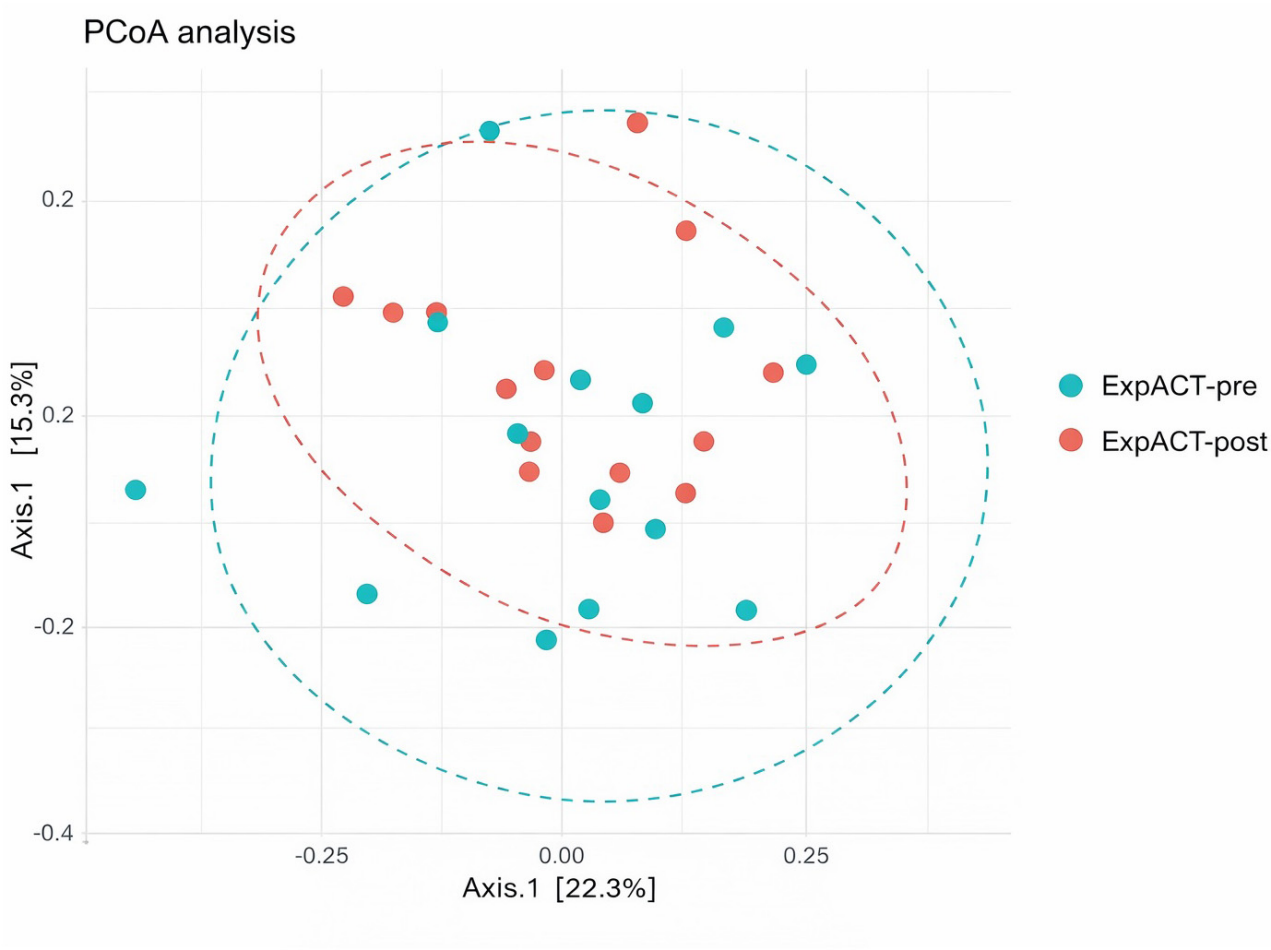
β-diversity of fecal microbiota at the species level, visualized by Principal Coordinates Analysis (PCoA) based on Bray–Curtis dissimilarity. The plot includes all samples from the ExpACT (ExpACT-pre, ExpACT-post) group following the probiotic supplementation. The X and Y axes represent the first two principal coordinates, explaining 15.3% and 22.3% of the total variance, respectively.

After probiotic supplementation, β diversity analysis using PERMANOVA revealed a significant shift in the overall microbial community structure between pre- and post-intervention samples (*R*^2^ = 0.0356, *p* = 0.041). The results are illustrated using PCoA (Figure 2). No significant changes were observed in α diversity metrics, measured by Shannon index and Chao1 index. A detailed overview of bacterial diversity indices is presented in Table 3.

**Table 3:** Gut microbiota composition, diversity indices, and metabolites during Phase 2: probiotic supplementation.

Variable	ExpACT-pre	ExpACT-post	*P*-value	*r*_rβ_
Shannon index	3.243 ± 0.250	3.279 ± 0.219	0.688	0.136
Chao1 index	539.379 ± 85.331	525.957 ± 78.193	0.551	0.181
*Bacteroidota*	19.138 ± 6.830	11.342 ± 7.059	**0.016**	0.733
*Lactobacillales*	0.955 ± 0.682	1.971 ± 1.513	**0.019**	0.714
*Lacticaseibacillus*	0.001 ± 0.002	0.151 ± 0.232	**0.003**	0.974
*Lacticaseibacillus casei*	0.001 ± 0.002	0.147 ± 0.223	**0.002**	0.974
*Lacticaseibacillus paracasei*	0.000 ± 0.000	0.004 ± 0.008	**0.028**	1.000
*Streptococcus thermophilus*	0.176 ± 0.178	0.871 ± 1.140	**0.009**	0.790
*Veillonellales*	1.158 ± 0.992	2.214 ± 2.619	**0.026**	0.676
*Blautia*	11.360 ± 4.889	15.157 ± 7.644	**0.048**	0.600
*Blautia schinkii*	4.950 ± 2.499	7.263 ± 4.672	**0.019**	0.714
*Lucifera butyrica*	0.153 ± 0.203	0.283 ± 0.332	**0.005**	0.848
*Parabacteroides*	1.544 ± 1.462	0.859 ± 1.010	**0.035**	0.638
*Phocaeicola vulgatus*	7.228 ± 3.981	3.807 ± 3.113	**0.013**	0.752
*Bacteroides thetaiotaomicron*	1.651 ± 1.947	1.091 ± 1.866	**0.022**	0.695
Methanol	1.61E-03 ± 7.86E-04	1.29E-03 ± 5.08E-04	0.055	0.581

Values are presented as mean ± standard error of the mean. Table summarizing significant differences (in bold) at *p* < 0.05 and effect size by rank-biserial correlation. r_rβ_, rank-biserial correlation; ExpACT, experimental group with probiotic supplementation.

**Figure 3:**
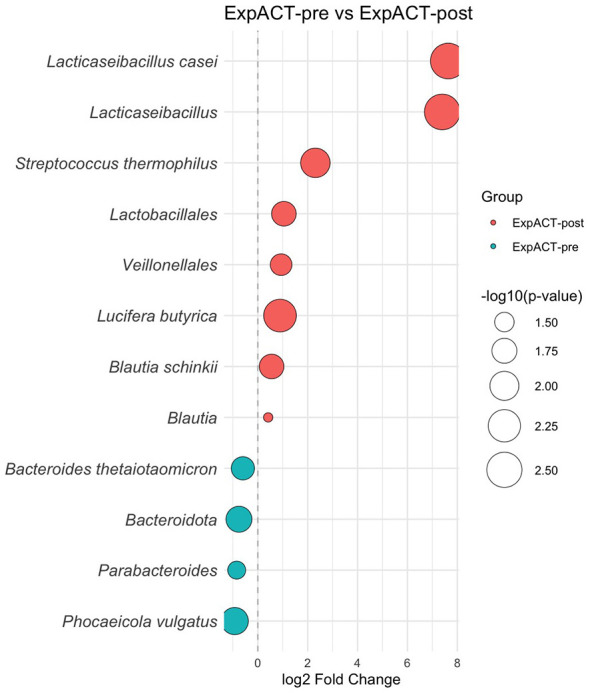
Differential abundance analysis (DAA) of gut microbiota after probiotic supplementation. Bubble plots display taxa across various taxonomic levels that were differentially abundant between respective timepoints in the experimental group after the probiotic supplementation (ExpACT-post vs. ExpACT-post). Blue circles indicate taxa significantly more abundant at the earlier timepoint, while red circles represent taxa enriched at the later timepoint. The size of each circle corresponds to the statistical significance of the difference, expressed as –log10 (*p*-value), based on a two-tailed Wilcoxon signed-rank test (*p* < 0.05).

Taxonomic analysis revealed several significant compositional shifts (Figure 3). At the phylum level, a significant decrease in the relative abundance of *Bacteroidota* was detected. Regarding lactic acid bacteria (LAB), a significant increase was observed in the order *Lactobacillales*, the genus *Lacticaseibacillus*, and the species *Lacticaseibacillus casei, Lacticaseibacillus paracasei*, and *Streptococcus thermophilus*. Furthermore, the order *Veillonellales* significantly increased following the probiotic intervention. In addition, significant shifts were detected in SCFA-producing bacteria, including increases in the genus *Blautia* and the species *Blautia schinkii* and *Lucifera butyrica*, as well as decreases in the genus *Parabacteroides* and the species *Phocaeicola vulgatus* and *Bacteroides thetaiotaomicron*. A detailed overview of bacterial composition is presented in Table 3.

#### 3.2.3 Stool metabolites

After probiotic supplementation, a trend toward a decrease in methanol concentration was observed (Table 3). Otherwise, no significant changes of other metabolites were observed. Interestingly, a significant positive correlation was detected between methanol and *Actinomycetota, Bifidobacterium*, and *Collinsella*. Moreover, a positive correlation was found between stool butyrate and relative abundance of *Faecalibacterium* (*r* = 0.521, *p* = 0.022).

## 4 Discussion

This study examined the independent effects of physical exercise and probiotic supplementation on gut microbiota composition, physical strength, and body composition in pediatric ALL patients in remission. The primary aim was to evaluate the impact of each intervention separately. We hypothesized that the physical exercise program would lead to favorable changes in body composition and muscular strength, while both the physical exercise program and probiotic supplementation would induce beneficial effects on gut microbiota diversity and the abundance of LAB and SCFA-producing bacteria. The main findings of the study are the following: (a) favorable changes in body composition and strength gains in patients completed physical exercise program; (b) a significant shift in overall microbial community composition based on β-diversity analysis following probiotic supplementation; (c) a significant increase in LAB bacteria and SCFA-producing bacteria after probiotic supplementation; and (d) a more pronounced modulatory effect of probiotic supplementation on gut microbiota composition compared with physical activity.

After the first phase (physical exercise program), both the experimental and control groups showed significant increases in body height and body weight, reflecting normal growth patterns in this pediatric cohort, which remained within the normal-weight range throughout the study period (World Health Organization, 2025). Interestingly, the increase in body weight in the experimental group was accompanied by a favorable, although non-significant, reduction in waist and hip circumferences. However, in the control group, both body weight and hip circumference increased significantly. Waist circumference is a sensitive indicator of central adiposity and cardiometabolic risk (Ross et al., 2020) and is particularly relevant in ALL survivors. The oncology population is predisposed to unfavorable body-fat distribution and increased metabolic and cardiovascular risk following treatment (Cebeci et al., 2024). Consistent with this, more than half of ALL survivors in the PETALE study (Lemay et al., 2019) who were 15 years post-remission, met criteria for obesity based on BMI, waist circumference, or body fat percentage. However, some studies have reported significant decreases in waist circumference after physical activity (Järvelä et al., 2012; Lopera et al., 2016), while others have reported no significant changes (Zhou et al., 2022) as observed in our study. Nevertheless, the absence of waist circumference increase in the physically active group suggests a potential protective effect of structured physical activity against central adiposity, possibly reflecting increases in fat-free mass rather than excess fat accumulation (Järvelä et al., 2012).

As expected, handgrip strength improved in children completed the 8-week physical exercise program. Handgrip strength is a widely used marker of overall muscular function in pediatric and other clinical populations (Bobos et al., 2020). However, findings across exercise studies in pediatric oncology survivors remain inconsistent, with some studies reporting significant improvements (Tanir and Kuguoglu, 2013; Braam et al., 2016) and others showing no measurable change in handgrip strength (Manchola-González et al., 2020; Lambert et al., 2021). Similar outcome variability has been reported for lower-limb power, such as the SJ and CJ (Braam et al., 2016, 2018), for which no significant improvements were observed in the present study. These heterogeneous results may reflect differences in training specifics, duration, intensity, frequency and baseline functional status. Importantly, reduced muscle strength and mobility can limit participation in age-appropriate physical activities and negatively affect overall quality of life (Marchese et al., 2021). Moreover, current evidence supports muscular strength as a marker of skeletal health in children and adolescents (Torres-Costoso et al., 2020). This is especially important in ALL survivors, who commonly demonstrate reduced bone mineral density as a consequence of treatment-related skeletal effects (Rohani et al., 2017). Therefore, even modest gains in muscular strength, as observed in our study, may have important clinical relevance in pediatric ALL survivors.

Following the physical exercise program, no robust or statistically significant changes in α or β diversity were detected in the present study. The Shannon index, however, showed a near-significant increase in the experimental group and a decrease in the control group, suggesting a possible trend toward improved microbial richness with physical activity. By contrast, our previous study demonstrated a significant increase in α diversity following combined physical exercise and probiotic supplementation in patients with ALL in remission (Bielik et al., 2023). This aligns with recent findings in adults, where higher levels of physical activity was associated with increased α diversity in colorectal cancer survivors (Himbert et al., 2022) and shifts in β diversity in breast cancer survivors (Paulsen et al., 2017).

Interestingly, following the physical exercise intervention, we detected an increased relative abundance of several beneficial SCFA-producing taxa, including *A. lactatifermentans, B. cellulosilyticus, C. eutactus*, and *P. rara*. The enrichment of these taxa is notable given that reduced *A. lactatifermentans* levels have been reported in multiple disease states, including ovarian (Mehra et al., 2023), pancreatic (Villani et al., 2024), and colorectal (Zhang et al., 2018) cancer. Moreover, *A. lactatifermentans* and *C. eutactus* have been linked to obesity-related metabolic profiles and cardiovascular risk factors (Lee et al., 2023). Additionally, higher levels of *Coprococcus* have been observed in physically active individuals (Bressa et al., 2017). A similar pattern was detected for *B. cellulosilyticus*, a taxon strongly associated with physical fitness measures. In the study by Jie et al. (Jie et al., 2021) taxa within *Bacteroides*, including *B. cellulosilyticus*, were consistently linked to greater exercise exposure and higher fitness scores. The increase in *P. rara* is also consistent with previous findings demonstrating a positive association between physical activity and increased *Prevotella* abundance (Petersen et al., 2017). Conversely, reduced *Prevotella* abundance has been described in newly diagnosed pediatric ALL patients (Liu et al., 2020).

After the physical exercise training, a significant reduction in the potentially toxic microbial metabolite TMA was observed. TMA, produced from choline and carnitine by gut bacteria, is associated with oxidative stress, inflammation, and impaired epithelial integrity (Romano et al., 2015). Lower TMA levels may also indicate reduced formation of trimethylamine N-oxide (TMAO), a procarcinogenic compound linked to colorectal cancer (Yang et al., 2022). Moreover, we detected a significant negative association between TMA and *Bifidobacterium*, consistent with evidence showing that *Bifidobacterium* spp. supplementation can restore gut dysbiosis and reduce TMAO levels by competitively inhibiting TMA-producing microorganisms or by directly metabolizing TMA (Zhou et al., 2025). Thus, the decrease in TMA may indicate favorable microbiome restoration in children after ALL treatment, consistent with findings by Cronin et al. (2018) following an 8-week exercise intervention.

As hypothesized, supplementation with the probiotic product was associated with significant changes in the gut microbiota structure of pediatric ALL patients in remission. Although changes in β diversity were statistically significant but modest, and no significant changes were observed in α diversity, several taxonomic shifts with large effect sizes were detected. The modest shift in diversity is common in probiotic interventions and could be explained by relatively short duration, single-product probiotic (Washburn et al., 2022) and clinical populations with high baseline variability (Chua et al., 2020). Similar to our findings, shifts in β diversity without parallel changes in α diversity have been documented in adults with functional bowel disorders (Bang et al., 2025) and in healthy adults (Ferrario et al., 2014). Interestingly, reduced interindividual variability was visible in the PCoA plots of β diversity, suggesting that the gut microbiota became more stable or constrained following supplementation (Hemarajata and Versalovic, 2013).

Notably, shifts in the dominant phylum *Bacteroidota* and increases in the relative abundance of health-associated genera, including *Blautia* (phylum *Bacillota*), indicate targeted modulation of the gut microbiota rather than global restructuring. These findings suggest that the probiotic intervention influenced specific microbial niches with functional and clinical relevance, while preserving overall community stability (Rodenes-Gavidia et al., 2023). The observed reduction of *P. vulgatus* (phylum *Bacteroidota*) following probiotic supplementation may further reflect a rebalancing of major commensal taxa, rather than a loss of core microbiota (Hemarajata and Versalovic, 2013). Comparable decreases in *P. vulgatus* have been reported in obesity-related microbiota interventions (Dewulf et al., 2013; Nicolucci et al., 2017), supporting the broader relevance of this pattern. In addition, the increase in *Blautia* is noteworthy, given its well-established links to SCFA production, gut barrier integrity, anti-inflammatory profiles, and more favorable metabolic and immune profiles. Similar probiotic-associated increases in *Blautia* have been described in previous oncology studies (Huang et al., 2023; Wang et al., 2025).

Additionally, the intervention led to enrichment of the order *Lactobacillales*, including probiotic-associated LAB (*L. paracasei* and *S. thermophilus*), as well as the closely related species *L. casei*. Previous studies have demonstrated that probiotic LAB generally do not persist in the gut and often disappear from fecal samples within 1 to 2 weeks (Alp and Kuleaşan, 2019). Nevertheless, as short-term ecosystem colonizers, they can still influence resident microbial communities. Previous *in vitro* and *in vivo* work (Button et al., 2023) has clearly shown that lactate-producing bacteria can cross-feed lactate to *Veillonella* spp. Therefore, LAB enrichment may have increased luminal lactate availability, creating favorable conditions for the observed increase in *Veillonellales*. Furthermore, this observation may help address the ongoing debate regarding *Veillonella* and clarify the relative contributions of probiotic supplementation and physical activity to the modulation of these bacteria. Previous studies (Scheiman et al., 2019) have highlighted *Veillonella* as a lactate-utilizing genus that increases in response to physical exertion, whereby exercise-induced lactate serves as a substrate for propionate production and has been linked to enhanced exercise capacity. Consistent with this, a previous study reported increased *Veillonella* abundance following fermented milk containing *L. paracasei* supplementation in patients with depression (Zhang et al., 2024). In this context, the findings of the present study indicate that probiotic supplementation was associated with a more pronounced effect on *Veillonellales* than physical activity alone, supporting the concept that probiotic-associated changes in nutrient availability and microbial interactions may provide an additional pathway for *Veillonella* enrichment.

A key strength of this study is the focus on a pediatric cohort of ALL patients in remission, a population in which gut microbiota research remains extremely limited. Whereas, most existing studies include individuals more than 15 years post-treatment, our cohort represents children within 5 years of remission, offering valuable insight into an understudied clinical window. Importantly, no adverse effects were reported following probiotic supplementation, which reinforces the safety of this approach and supports further evaluation in similar pediatric oncology populations.

However, this study also has several limitations. First, although significant changes in microbial composition and diversity were identified, clinical outcomes were not assessed in parallel, limiting interpretation of functional or health-related impacts. Second, reliance on NMR-based stool metabolite profiling may not fully capture the metabolic complexity of the gut ecosystem. Third, dietary energy intake and habitual physical activity outside the intervention were not recorded. Fourth, a placebo control group was not included during the probiotic supplementation phase, and because this phase followed the physical activity intervention within the same group, the observed effects cannot be considered fully independent of the preceding intervention and may also reflect time- or remission-related changes. Fifth, differential abundance analyses were performed on relative abundance data without applying compositional data transformations, therefore the reported *p-values* should be interpreted as exploratory. Finally, the sample size was modest, and data collection occurred during the winter season, when gut microbiota composition may be subject to seasonal variation, which could have influenced the results. Nevertheless, despite these limitations, the study provides valuable insights into microbiota modulation and lifestyle-related interventions in pediatric ALL patients in remission.

## 5 Conclusion

In this two-phase, controlled trial of pediatric ALL patients in remission, 8 weeks of strength training were associated with increased handgrip strength and favorable shifts in body composition, while inducing only minimal changes in gut microbiota composition. In contrast, 8 weeks of supplementation with the probiotic product resulted in targeted modulation of specific gut microbial taxa, including increases in short-chain fatty acid–producing bacteria, alongside significant but modest alterations in β diversity. Collectively, these findings suggest more pronounced microbiota-related effects during the probiotic supplementation phase, whereas physical activity primarily contributes to improvements in muscular strength and maintenance of body composition in pediatric ALL survivors in remission.

## Data Availability

The data presented in the study are deposited in the GenBank repository, BioProject accession number PRJNA1440226.
